# How can more women with pre-existing type 1 and type 2 diabetes be supported to prepare for pregnancy after a baby loss? A qualitative exploration of lived experiences in the UK

**DOI:** 10.1136/bmjopen-2023-083192

**Published:** 2025-01-15

**Authors:** Eleanor Dyer, Ruth Bell, Ruth Graham, Judith Rankin

**Affiliations:** 1 Population Health Sciences Institute, Newcastle University, Newcastle upon Tyne, UK; 2 Geography, Sociology and Politics, Newcastle University, Newcastle upon Tyne, UK

**Keywords:** QUALITATIVE RESEARCH, Diabetes in pregnancy, Health Services

## Abstract

**Abstract:**

**Objectives:**

Explore, understand and analyse how women with pre-existing diabetes can be better supported during the inter-pregnancy interval—the time after a baby loss and before a subsequent pregnancy.

**Design:**

Qualitative, semi-structured online interviews took place between November 2020 and July 2021. Data were analysed using Reflexive Thematic Analysis.

**Setting:**

Participants reflected on care received at primary and secondary centres across the UK.

**Participants:**

Twelve predominantly White, British women with type 1 (n=9) and type 2 (n=3) diabetes with experience of baby loss and subsequent pregnancy were recruited through social media.

**Results:**

Three interrelated themes: (1) decisions around becoming pregnant after baby loss, (2) the triple burden of baby loss, diabetes and preparing for pregnancy, (3) gaps in the inter-pregnancy interval. Most (n=10) participants wanted to become pregnant again as soon as possible. The short inter-pregnancy interval (median=7 months) highlights a potentially small window of opportunity to support women to grieve and prepare for pregnancy. Providing timely access to care and support in the inter-pregnancy interval without overburdening women might be challenging due to structural issues in services and gaps in referral pathways.

**Conclusion:**

Women with pre-existing diabetes may experience challenges in accessing appropriate pre-pregnancy care in the inter-pregnancy interval. Our findings suggest that one-size-fits-all approaches are likely to be less effective in meeting diverse needs of this group and that more personalised, targeted support is needed. All healthcare professionals across the different parts of the care provision structure need greater awareness of the issues faced by this group to maximise timely access to the appropriate pre-pregnancy care and support.

**STRENGTHS AND LIMITATATIONS OF THIS STUDY**

- The qualitative approach using semi-structured interview methods allowed for a detailed exploration of this sensitive topic.
- This paper represents the experiences of women with pre-existing diabetes from across the UK who had experienced a range of baby losses, highlighting differences in healthcare services delivery.
- There was no limit on time elapsed since the events women were reporting on, which could have impacted participant recollections, and care provision could have since changed.
- The women with diabetes participants predominantly had type 1 diabetes (n=9), so the voices of women with type 2 diabetes (n=3) were underrepresented.
- The small sample provides valuable in-depth analysis of women’s experiences, but consequently does not seek to represent the views of all women in this situation, for example, women who did not pursue a subsequent pregnancy after baby loss.

## Background

Pre-existing diabetes is the most prevalent chronic condition affecting pregnancy in the UK.^1^ Studies suggest 1–2% of pregnancies^2^ are affected by type 1 (T1DM) and type 2 (T2DM) diabetes annually and the numbers have risen in recent years.^3 4^ This increase is attributed to both the rise in overweight and obesity rates in the general population, which frequently leads to T2DM,^5^ and the increased prevalence of pregnancies in older women.^3 5 6^

Diabetes in pregnancy poses risks to both mother and child, increasing the risk of baby loss (miscarriage, stillbirth, neonatal death and termination of pregnancy for medical reasons), congenital anomalies, premature delivery and being large for gestational age.^7^,^10^ Although the pathophysiology and aetiology of T1DM and T2DM are distinct, they are associated with similar rates of risk^4^ and are managed, clinically, in a similar way before and during pregnancy.^5^

To reduce risks, women with diabetes (WWD) are advised to plan and prepare for pregnancy by taking high-dose folic acid, optimising blood glucose control and stopping teratogenic medication.^5 11 12^ However, as there is no standardised care pathway in the UK, provision is patchy and variable across services, and it is unclear who is responsible for supporting WWD’s preconception health in the inter-pregnancy interval.^13^ The 2023 National Pregnancy in Diabetes (NPID) Audit reported that only around one in eight women were ‘well prepared’ for pregnancy, with no improvement over the past 9 years.^4^ The reasons for this are complex and not fully understood. The limited research suggests approximately 50% of WWD do not seek support from healthcare services to prepare for pregnancy,^3 14^ even after experiencing baby loss.^10 15^ Very little is known about WWD experiences of becoming pregnant again after a baby loss.

Although WWD are more likely to experience a baby loss, there is a gap in understanding how best to support this group to prepare for a subsequent pregnancy. This exploratory study is the first to focus specifically on WWD lived experiences of becoming pregnant again after a baby loss. The findings connect under-researched areas seldom explored together and provide new insight into potential reasons why WWD do not ‘optimally’ prepare for pregnancy after experiencing a baby loss, highlighting ways to improve inter-pregnancy care.

## Methods

### Research question

How can women with T1DM and T2DM be better supported to prepare for pregnancy after a baby loss?

### Research aim

This qualitative study aimed to explore the lived experiences of preparing for pregnancy after baby loss among women with pre-existing T1DM and T2DM to better understand the challenges faced by this group and how healthcare services could better meet their needs.

### Design

Thirty in-depth, semi-structured interviews with 12 WWD (T1DM=9, T2DM=3) and 18 healthcare professionals were conducted to better understand the challenges in supporting WWD after a baby loss. The findings presented here provide the perspectives from the 12 WWD. The findings from the 18 healthcare professionals are reported elsewhere.^13^ The approach used to report the methods and findings was informed by the Standards for Reporting Qualitative Research (SRQR).^16^

### Participants and recruitment

Participants across the UK were purposively recruited through social media (Facebook=6, Mumsnet=1 and platform X=5 [previously known as Twitter]). Recruitment continued until ‘meaning saturation’ was achieved^17^ whereby the richness and complexity of the data were deemed adequate in terms of addressing the research question,^18^ a decision made during the latter stages of data collection.

### Patient and public involvement statement

To ensure the interviews explored issues that were relevant to WWD who had experienced a baby loss, the Stillbirth and Neonatal Death (SANDS) Charity was able to advise on the content to ensure terminology and questions were phrased sensitively. Three women with lived experience of diabetes and baby loss were also involved in piloting the topic guides for the interviews which gave us confidence that the topic was relevant and needed and that the sensitive nature of the topic was handled appropriately. WWD have also been involved in providing feedback on the findings and co-developing materials for the wider dissemination of the findings among patient communities, for example, by choosing what findings are shared, when, and in what format.

### Data collection

Twelve WWD took part in an in-depth, semi-structured telephone or video interview with the first author (ED) between November 2020 and July 2021. Interviews ranged from 44 to 65 min (average length=59 min) which allowed for in-depth, nuanced and rich data. Interviews were informed by a topic guide developed with input from the SANDS charity (table 1). The interview focused on the inter-pregnancy interval, namely, the period between baby loss and the start of a subsequent pregnancy. Key aspects covered in the interviews included the impact of complex emotional events on satisfaction with care, interpretations of the reasons for the baby loss, and how best to support parents making decisions about a subsequent pregnancy. The topic guide was revised throughout data collection in response to emergent findings regarding the prompts used and questions of interest that came to light. This was consistent with the social constructionist underpinnings of the study and the recognition that knowledge creation is co-constructed.^19 20^ An overview of participants’ demographic information is provided in table 2.

**Table 1:** Topic guide

Introduction	
Thank you for seeing me today and offering to take part in this study. Confirm receipt of signed informed consent form and project information sheet. Recap project information sheet and record verbal consent (this will be audio recorded so my supervisors can witness informed consent if needed). You do not have to answer all the questions if you do not want to. Let me know if you want to have a break or stop the interview at any point. Feel free to ask questions at any stage during the interview.	
Topics/Prompts (N.B. This is not a rigid interview schedule. This topic guide was intended as a reminder of topics to talk about and includes a list of prompts in the event they are needed, for example, if the participant does not say very much).	
1. I am so sorry to hear about the loss of your baby/your baby’s death. Would you like to tell me a little about your baby? (What was their name? When did it happen? What happened? How did you feel at the time? How do you feel now?)	
2. Thank you for sharing that with me. I will not be asking questions specifically about your pregnancy (or neonatal care) with your baby (insert name if applicable), but about the time after your baby died and before becoming pregnant again. However, please feel like you can talk about your baby or that pregnancy, if you want to, at any time during our interview.	
3. Thinking back to the time between your baby dying and becoming pregnant again: How long was the gap between your baby dying and becoming pregnant again? How did you feel about this at the time? How do you feel about this in hindsight? How did you feel about becoming pregnant again at the time? How do you feel about it now, looking back?	
4. After your baby died, and before you became pregnant again, did you receive/were you offered any bereavement support?	
If Yes	If No
What sort of care did you get? Where did you get this care? How did you find out about it? Do you feel it helped you in terms of feeling ready to become pregnant again? In what ways? Did you feel your needs at the time were met or was there more that could have been done to help you (both at the time and in hindsight)?	Did you want care, but it wasn’t offered/you weren’t able to access? How did you feel about this at the time? How do you feel about this in hindsight? Is there anything that stopped you from getting bereavement care?
5. Can you tell me a bit about your diabetes? What type of diabetes do you have? How long have you had diabetes? In what ways does it affect your life?	
6. Thinking about the time between your previous pregnancy and the next pregnancy, did you receive any/were you offered any pre-pregnancy care or advice from healthcare professionals to help with becoming pregnant with diabetes?	
If Yes	If No
What sort of care/advice? From whom? How long before pregnancy? How did you feel about this at the time? Did it mean more visits to the doctors/hospital? How many different healthcare professionals did you speak to in the time between your previous pregnancy and the next pregnancy? Can you tell me about some of the conversations/interactions you had with healthcare professionals? Did you do anything differently when preparing for the next pregnancy? What sort of things? How did you feel about these conversations/interactions? At the time? In hindsight? What aspects worked well? Less well? Did you feel your needs were met at the time or was there more that could have been done to help you? What aspects worked well and what worked less well? In what ways was your previous pregnancy acknowledged or taken into account?	Did you want care, but it wasn’t offered/able to access?—How did you feel about this at the time? How do you feel about this in hindsight? Did you look for any advice/support from elsewhere? Can you tell me a little about what/where? Is there anything that would have improved the likelihood of you attending Pre-Pregnancy Care (PPC)?
7. Overall, do you feel you were able to access the care and support that you needed before becoming pregnant again? Was there anything that could be done differently that would have helped you? Would you have liked more support? What type of support would have been helpful? Would you have liked more advice or information? What type of advice/information would have been helpful? Where is the best place for you to access this advice/information? (if relevant) Do you feel your ethnicity has affected the care you were able to access? What could be done to improve access for you?	
8. Anything else that the interviewee/interviewer feels has been missed/did not get a chance to discuss fully.	
9. Any questions or is there anything else you would like to talk to me about?	
End of interview.	
10. Thank you for taking the time to talk to me today and for taking part in my research—Next steps (eg, I will send you an e-mail with some helpful information and links).	

PPCPre-Pregnancy Care

**Table 2:** Participant demographics

	Women with diabetes
Type of diabetes	Type 1=9
	Type 2=3
Age at the time of the interview	28–50 (median=36) years
Age at diagnosis type 1 diabetes	7-28 (median=11) years
Age at diagnosis type 2 diabetes	15-32 (mean=26) years
Age at first pregnancy	23-35 (median=29.5) years
Type of baby losses reported (four participants reported multiple losses)	Early miscarriage (<12 weeks pregnant)=8
	Late miscarriage (12–24 weeks pregnant)=5
	Stillbirth (24+weeks pregnant)=2
	Neonatal death (death in the first month of life)=3
Ethnic background	White=11
	Black/African=1
Education level	Further education=1
	Undergraduate degree=4
	Postgraduate degree=6
	Undisclosed=1
Relationship status at the time of the interview	Married=8
	Relationship=2
	Divorced=1
	Single=1
Location during inter-pregnancy interval	London=3
	Northeast England=3
	West Midlands=2
	East Midlands=1
	Northwest England=1
	Yorkshire and Humber=1
	Southeast England=1
Employment status at the time of the interview	Employed full-time=11
	Retired=1

### Data analysis

**Figure 1:**
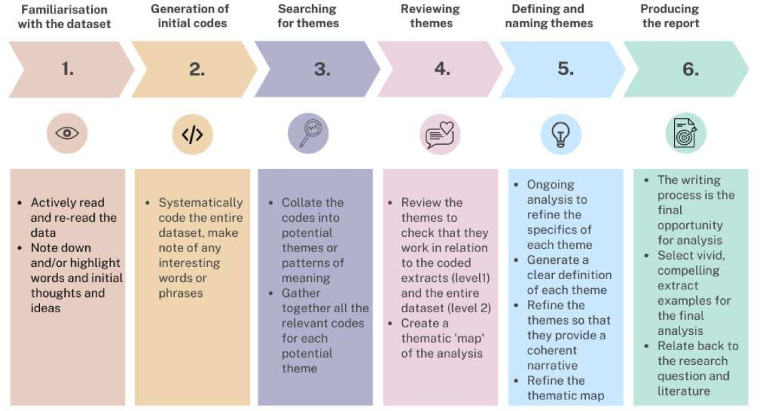
An overview of Braun and Clarke’s (2006) six-step phases of Thematic Analysis.

The audio recordings from the interviews were manually transcribed verbatim by the lead author (ED) and a clerical officer. The anonymised transcripts were coded and analysed using Braun and Clarke’s Reflexive Thematic Analysis^21^ which builds on the six, iterative steps used in Thematic Analysis^22^ and is summarised in figure 1. Reflexive Thematic Analysis embraces the interpretive role of the researcher, and acknowledges that engagement with the dataset and the generation of themes is mediated by the researcher’s values, skills and experience.^21^ The coding and analysis steps were led by the first author (ED) and were facilitated with qualitative analysis computing software (Quirkos).^23^ The transcripts were read multiple times before subsequent analysis using a social constructionist interpretive framework. Social constructionism suggests that no stable and objective reality is waiting to be discovered, but that reality is a product or ‘construct’ of human activities.^24^ As such, reality is co-constructed between the researcher and the participants, and the meaning of experiences is shaped and interpreted by individuals,^19 25^ and allows researchers to seek out complexity of views directly from participants.^19^ Social constructionism has been an influential theoretical position in the sociology of health and illness since the 1980s, and is recognised as a well-established approach to qualitative health research.^24 26 27^

Data analysis was inductive and focused on identifying themes generated from the interviews. Three experienced researchers (RB, RG, JR) were also involved in the data analysis by independently reviewing the codes, and interpretations were discussed at monthly data meetings. A Research Manager from SANDS was involved in the final data meeting, where agreement was reached on the key themes, subthemes, illustrative quotes and participant identifiers.

## Results

**Figure 2:**
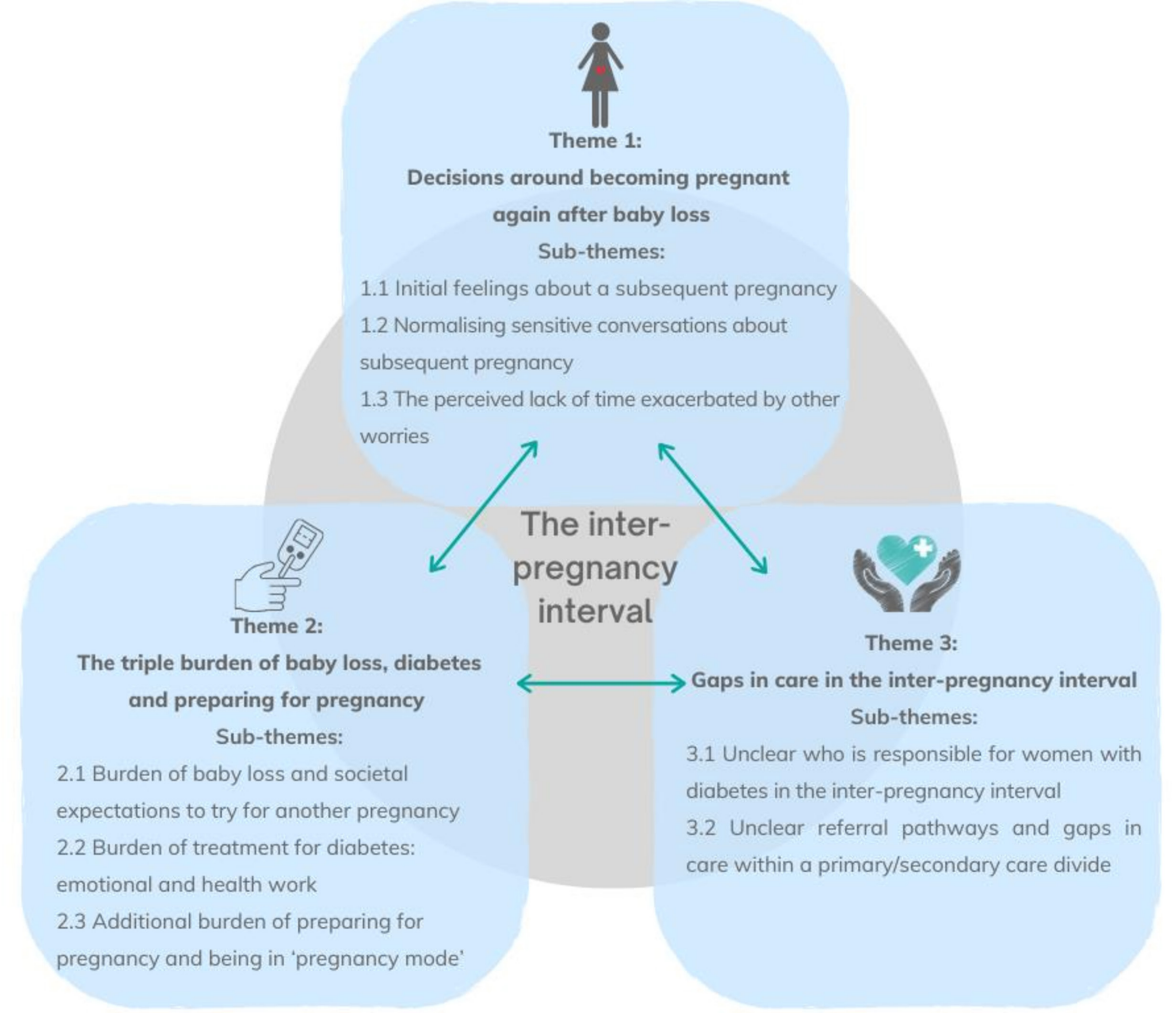
A visual overview of the themes and sub-themes.

The analysis generated three main themes: (1) decisions around becoming pregnant after baby loss, (2) the triple burden of baby loss, diabetes and preparing for pregnancy, (3) gaps in care in the inter-pregnancy interval. The themes and sub-themes are set out, below, along with illustrative examples from the data. See figure 2 for a visual overview of the themes and subthemes, illustrating how the themes interact with each other.

### Theme 1: decisions around becoming pregnant again after baby loss

The first theme represents the time point directly after the baby loss, reflecting participants’ initial thoughts about a subsequent pregnancy, before any active planning.

#### Initial feelings about a subsequent pregnancy

All 12 participants knew straight away that they wanted to try for another baby at some point.

We’d got our head around having a child, emotionally. Yes, I was ready to become a mum (but) going through what we had gone through (again) I wasn’t ready (for that). WWD6 T1DM Late Miscarriage

I (wanted to try sooner) but my body was not allowing me because … I had this never-ending period. WWD9 T1DM Stillbirth

**Figure 3:**
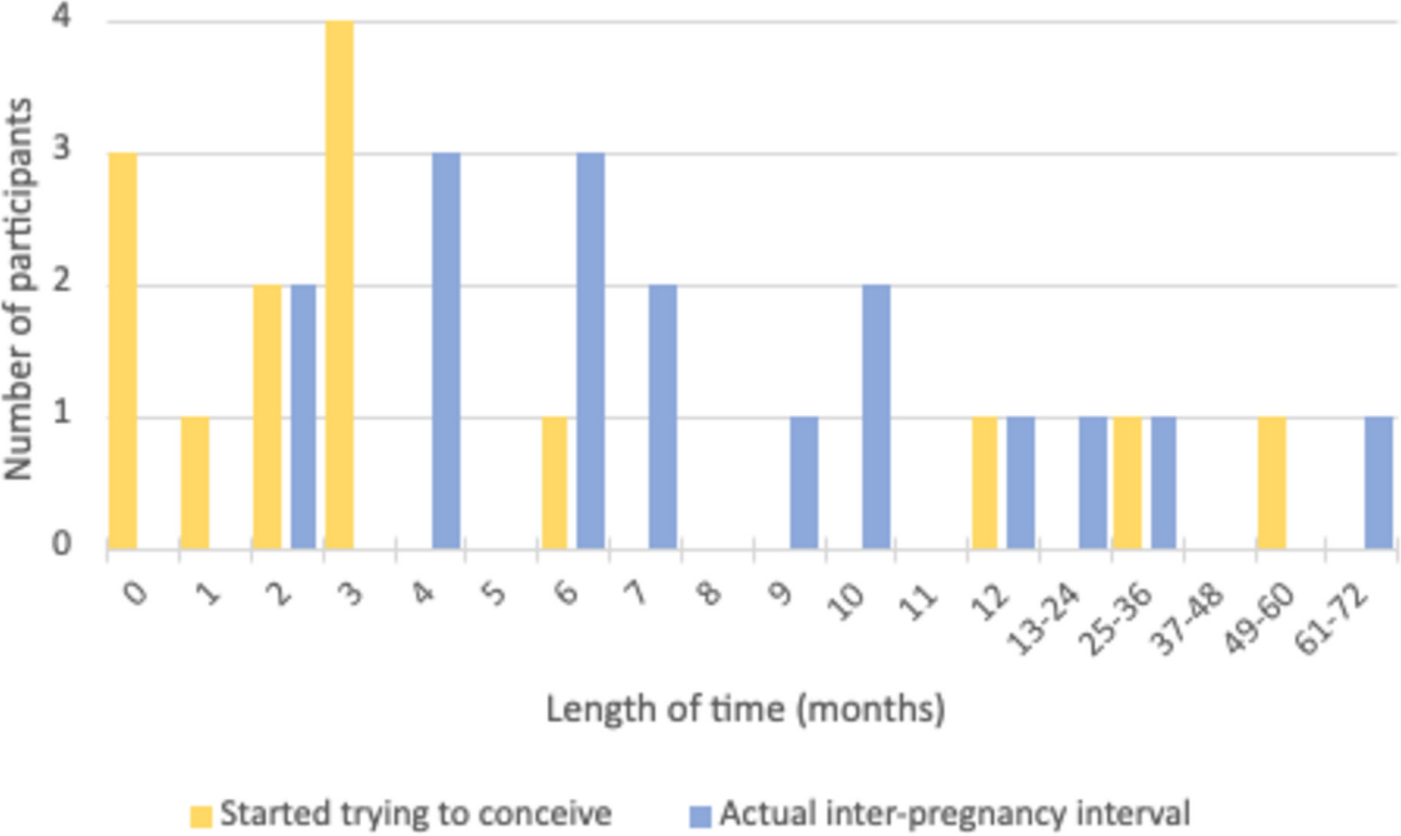
The length of inter-pregnancy interval as reported by participants.

The inter-pregnancy intervals ranged from 2 to 66 months (median=7 months), as illustrated in figure 3. Ten of the 12 participants started trying for subsequent pregnancy within 6 months of their baby losses. The two participants who waited longer than 12 months experienced later losses (stillbirth and neonatal death) and had older children to care for, which were factors in the length of time they waited (36 months and 66 months, respectively).

I felt very much that it was my responsibility to make sure that (the older child and family) was alright. But it was incredibly stressful and from the outside it might have looked like I was fine … but obviously it wasn’t fine. WWD7 T2DM, Neonatal Death

#### Normalising sensitive conversations about subsequent pregnancy

The participants tended to welcome the opportunity to discuss future pregnancy plans. However, they would not always feel comfortable initiating that conversation themselves as it felt ‘a bit odd’ telling healthcare professionals about their intention for a subsequent pregnancy (WWD9).

I’m not really superstitious, but do you want to tempt fate by … saying, ‘ I want to get pregnant’ … or do you just think, ‘ let’ s just go for it and just see what happens.’ … The idea of having that conversation with my GP, it would never have happened. WWD3 T1DM, Multiple Early and Late Miscarriages

Several participants spoke about how they would have appreciated it if a trusted healthcare professional could have *‘*taken the lead*’* (WWD4) to reach out just to ask, ‘How are you doing?’ (WWD4) to open up a conversation about whether there was any additional support that could be offered.

As evident in subtheme (1.1), for the conversation to be timely, it may be required shortly after the baby loss. However, participants reported that conversations about subsequent pregnancies were not normalised and were not necessarily initiated by healthcare professionals.

The consultant had said, ‘ I hope to see you in future pregnancies’ …. Thinking about it now [ preparing for the next pregnancy ] should have been [ mentioned ], but at the time it wasn’ t. WWD6 T1DM, Late Miscarriage

WWD6 was not provided with any advice or information about a subsequent pregnancy during the inter-pregnancy interval, and the conversation was closed rather than left open. Preparing for pregnancy was not mentioned, and WWD6 was advised that only when they were actually pregnant would they be able to access support.

Some participants were advised by healthcare professionals to wait a specified period of time before trying to conceive, to allow time to grieve, heal emotionally and physically and, in some instances, to allow time to receive post-mortem results. There were mixed reactions to the advice to wait, and even those who could see the benefits of waiting found it upsetting. A recurring theme was how hard it was to balance the ‘yearning to have a baby’ (WWD7) with grief and the medical advice to wait for a subsequent pregnancy.

Participants tended to see the benefit of building trusting relationships with healthcare professionals, and continuity of care was largely recognised as beneficial to facilitating candid conversations about pregnancy plans.

I really saw the benefit of [ continuity of care ] just seeing one person, and they know your history, and it’ s easy … I didn’ t have to go and explain everything. WWD9 T1DM, Stillbirth

#### The perceived lack of time exacerbated by other worries

The decision to try to become pregnant again was further complicated for many of the participants who had other considerations on top of their diabetes. Increasing age was a recurring concern. For example, WWD2 felt like there was a ‘ticking clock’ and worried that they would become too old to conceive or that their diabetes ‘will progressively get worse’ (WWD2):

We were trying pretty much straight away … I'm 36 this year which feels like ancient in child- rearing age … I feel like if I get past 40, it’s not going to happen. WWD2 T2DM Neonatal Death

Co-occurring fertility issues may have added an additional layer of pressure. Not knowing how long it would take to conceive or whether the subsequent pregnancy would be ’successful' made some participants try sooner than they might otherwise have.

We were not expecting for it to happen so quickly. The first one took a year and a half, and the second one was really quick, but who knows what’s going to happen next time …, so let’s start now. WWD12 T1DM after Early Miscarriage

A recurring motif was how lengthy the inter-pregnancy interval seemed for participants, even though, in hindsight, it was not. This was captured by WWD10, who, when asked about how they felt at the time about the interval of time between their miscarriage and becoming pregnant again, replied:

It was grim. Objectively … it was 4 months and feels like it was years … I remember at the time being like, ‘something is definitely wrong with me, I can’t get pregnant.’ … There was only 2 months of trying before I then got pregnant again, but that to me felt like absolutely aeons of time. WWD10 T1DM, Early Miscarriage

For some participants, the decision to become pregnant was compounded by a need to be pregnant again. For example, some of the women spoke of being ‘obsessed’ (WWD1) and *‘*desperate’ (WWD3) to be pregnant again.

I really wanted to get pregnant from the day my milk came in … As soon as my milk came in, I was like, ‘I need a baby, my body needs a baby.’ WWD9 T1DM, Stillbirth

For some participants, having a baby was reported as the only way to heal their pain, and the overwhelming urge to become pregnant again was stronger than the need to be mentally or physically ready.

I don’t believe I grieved for (my baby) in those ten months. I was grieving, but I hadn’t come to terms with it …. All I needed was another baby to be growing inside … I thought this other baby is going to take all this away. This baby is going to fix my heart …. This will make it right, and I can stop the pain if I know I’m pregnant. WWD8 T1DM, Neonatal Death

There was a contradiction here in women’s accounts of their experiences: WWD reported feeling like they did not have enough time whilst simultaneously feeling like it was taking too much time to become pregnant again, indicating the complexity of how the participants experienced time in this context.

### Theme 2: the triple burden of baby loss, diabetes and preparing for pregnancy

The second theme exposed how WWD may face intersecting burdens in the inter-pregnancy interval. Participants were asked to reflect on what it was like to live with diabetes, specifically about their experience managing their condition in the inter-pregnancy interval. Participants spoke of busy lives, with work and family commitments to manage. Some also looked after older children or relatives.

#### Burden of baby loss and societal expectations to try for another pregnancy

Participants reported that healthcare support in the inter-pregnancy interval tended to focus on the physical aspects of their diabetes management, and they were not routinely referred to counselling services to help them process the emotional or psychological aspects of baby loss unless they initiated it themselves. Some participants described how good-intentioned societal expectations to ‘just try again’ (WWD7) were unhelpful and distressing, as it was not that straightforward with diabetes. WWD7 spoke about how people would say things out of awkwardness, and the following example stuck with her:

I felt like there was a pressure from society and … people are incredibly blunt … and just say ridiculous things about having babies and … that can be distressing to women …. People would say, ‘You are going to have another one, aren’t you? You’re not going to let this beat you?’ And I … found that a really incredibly odd thing to say …. I found it really hard being on maternity leave and seeing other people with babies. WWD7 T2DM, Neonatal Death

#### Burden of treatment for diabetes: emotional and health work

WWD1, who had T1DM, spoke of how maintaining tight control of her diabetes required constant effort and planning, especially around work commitments.

Just, ‘what am I going to eat?’ took a lot of effort …. The more effort I put in, the better the results are, so it’s not something that just runs smoothly and is fine, and I can just ignore it … If I do that, the then levels always end up being slightly higher than I would like them to be. WWD1 T1DM, Early Miscarriage

For some WWD, despite considering healthcare professionals to be empathetic, they did not feel like they understood what it was like to live with their condition, especially as each individual uniquely experienced diabetes.

Some participants commented that psychological support in the inter-pregnancy interval was beneficial in better understanding the emotional work required in managing their condition.

The thing that helped with the diabetes (was the psychologist) because he specialised in diabetes. He was working at the clinic, he knew a fair amount about diabetes in pregnancy and was like, ‘you’re right that this is going to be really hard …, you do need to be really controlled, it’s not you being over the top about it’, that sort of thing, which really helps. WWD10 T1DM, Early Miscarriage

Technology, such as ‘Libre’ sensors or Continuous Glucose Monitoring, was cited by some participants (predominantly T1DM) as beneficial for maintaining tight control of their blood glucose levels without further increasing the burden.

#### Additional burden of preparing for pregnancy and being in ‘pregnancy mode’

The majority of participants commented on how being ‘optimally prepared’ for pregnancy was not straightforward and took a great deal of effort. A powerful theme generated from the analysis was how being ‘optimally prepared’ for pregnancy, as per the NICE NG3 guidelines,^5^ required women to act as if they were pregnant, which WWD1 (T1DM) termed ‘pregnancy mode’. The burden of being in ‘pregnancy mode’ meant that some participants did not want to prepare for pregnancy ‘for longer than I have to’ (WWD10) owing to the intensive effort it involved. For these participants, the burden of being in ‘pregnancy mode’ meant it was unrealistic to be ‘pregnancy ready’ on the off-chance that they fall pregnant.

I essentially had to convince myself every month that it was very possible that I was pregnant … I had to believe that until I get my period and then be like, okay, I'm not … I found that really challenging […] if I was in that cycle for a year or something, I just don't know that I could keep it up. WWD10 T1DM Early Miscarriage

WWD1 commented on how preparing for pregnancy with T1DM ‘takes over your life’ (WWD1) and likened the level of control required to having an eating disorder because blood glucose levels were the ‘one thing I can control’ (WWD1). Similarly, WWD10 described the ‘really frequent testing’ and ‘very rigid behaviours around food’ required for her to be optimally prepared for pregnancy.

WWD10 noted that ‘pregnancy mode’ could, at times, feel like ‘an exercise in futility’, whereas her friends did not face this burden when trying for a baby.

You’re putting so much effort into something, and chances are it doesn’t matter, because chances are you aren’t pregnant. … Just setting your alarm every night right to wake up at two in the morning and test your blood. … The reality is if I’m doing that, we are trying (to get pregnant) … it felt like much more of an on and off switch … we’re either trying, or we’re not trying …. I certainly have friends who are like … ‘I’ve just decided to come off that pill, and we’d see what happens’, which just feels like such a luxury. WWD10 T1DM, Early Miscarriage

All women interviewed took the responsibility of preparing for pregnancy themselves. The WWD who participated were generally interested in the research topic and, therefore, had good knowledge of the NICE NG3 preconception guidelines^5^ and available services in their area at the time of interview. However, the participants had differing baseline knowledge about the benefits of pre-conception care. For example, pregnancy was not mentioned to WWD2 when she was diagnosed with T2DM 1 year before her first pregnancy and only found out that preparing for pregnancy could reduce the risk of baby loss after she experienced a neonatal death.

Most participants noted that they would have appreciated more holistic support in the inter-pregnancy interval, as it was a mentally and physically challenging time for them. For women with T2DM, preparing for pregnancy may involve managing their condition in an entirely new way, and they may not be aware of what is required if they are reviewed infrequently.

Some participants did not find pre-pregnancy care told them anything they did not already know about the need to prepare for pregnancy, physically, from a diabetes point of view. Others welcomed pre-pregnancy care as it allowed them to gain access to technology to help them better manage their condition, so they found it more useful.

Nothing much really changes, except with the Libre …. You’re still only seen every 6 months, and you get advice on what they can see. I’m like, ‘Okay, I was already doing that, I’m already aware of the folic acid. WWD9 T1DM, Stillbirth

### Theme 3: gaps in care in the inter-pregnancy interval

The third theme centres on women’s perceptions of potential gaps in their experiences of care during the inter-pregnancy interval. Participants reported finding services a challenge to navigate and so it was burdensome to access the care or support they needed. There were also apparent disparities between the accessibility and provision of care for women with T2DM compared with those with T1DM.

#### Unclear who is responsible for women with diabetes in the inter-pregnancy interval

Participants reported finding themselves at the intersectional margins of baby loss, diabetes and preparing for pregnancy. Without wholly belonging to any one group, no one healthcare area had an overarching responsibility to care for the needs of this group.

I think the hospital thought (the local charity) is dealing with her, and there was absolutely nothing after (my baby died), which really, I really needed. WWD8 T1DM Neonatal Death

This seemed to be the case for women with T2DM in particular, where participants commented that practitioners in primary care tended to assume that women were being offered support in secondary care and vice versa. WWD2, for example, was told by her doctor that someone from the hospital ‘should be in touch with you about (therapy)’, but no one was. When WWD2 brought it up with the doctor again, the only support offered was a general ‘talking therapies’ service. A recurring theme from participants questioned the appropriateness of a *‘*beating the blues’ (WWD7) type service aimed at general mental health and not for those who had experienced a bereavement or trauma complicated by diabetes.

On the whole, this meant participants did not find it straightforward to access pre-pregnancy care that was equipped to support them holistically. For example, participants talked about how physical diabetes support did not support the psychological side of diabetes, and bereavement or grief support was unequipped to consider the context of diabetes.

The bereavement midwife was brilliant. But she wasn't specialised in diabetes … it’s very, very much about you've lost a baby, but they don't deal with the fact that you've lost a baby and your health condition could be that reason WWD5 T1DM Late Miscarriage

WWD6 recalled how she missed out on pre-pregnancy care in the inter-pregnancy interval.

He did say I should be under a team (and) that was literally how it was left. But it wasn’t something that was massively pushed … thinking about it now it could have been. … (when) I found out I was pregnant … (I made) a phone call back to the diabetic nurse saying, ‘look, I’m pregnant like what do I do?’ WWD6 T1DM, Late Miscarriage

Not knowing who was responsible seemed to result in WWD falling into gaps in care provision, or experiencing inconsistencies in advice.

#### Unclear referral pathways and gaps in care within a primary/secondary care divide

After experiencing a baby loss, the care of the WWD in this study was transferred back to whatever care they had before the pregnancy. Generally, the participants with T1DM were predominantly managed in secondary care and T2DM in primary care with support from specialist services. Participants described unclear referral pathways in the inter-pregnancy interval as gaps into which they could fall.

This period when you’re trying, but not pregnant, or don’t know whether you’re pregnant, where you still technically fall under your normal diabetes team, but they don’t really … think of the pregnancy stuff … that’s not really something that they’re set up to deal with, and the obstetricians aren’t set up to deal with you until you’re actually pregnant. So, I think there’s a little gap in that pathway. WWD10 T1DM, Early Miscarriage

Participants reported a great deal of variation in the types and availability of services. In general, participants spoke about being required to be responsible for proactively seeking out and initiating referrals from their GP or Diabetes Specialist Nurse to another service, for example, for bereavement, mental health or pre-pregnancy support.

It went back to … the yearly appointments where they check your feet and do the HbA1c check and everything like that …. When I spoke to the doctor and said, ‘I’d like to get pregnant, what can I do about my diabetes?’ that was me asking. No one specifically spoke to me about that. WWD2 T2DM, Neonatal Death

In some services, it was possible for the participants to self-refer to pre-pregnancy care, which made it easier for them to access timely care. However, women with T2DM reported being more reliant on being referred to pre-pregnancy care and specialist diabetes services by their GP. Sometimes, the pre-pregnancy clinic was held in the same location as the antenatal clinic, which may make sense in terms of service provision but was distressing for some participants.

## Discussion

This exploratory study is the first to focus specifically on the lived experiences of preparing for pregnancy after baby loss among women with pre-existing T1DM and T2DM. All participants knew straight away that they wanted to try for another pregnancy at some point in the future. Thoughts and feelings around having a subsequent pregnancy may happen sooner than healthcare professionals might assume. A short inter-pregnancy interval means WWD may not have much contact with healthcare professionals, so there might not be much time to support them in preparing for a subsequent pregnancy. A recurring theme was the tension WWD described in balancing the yearning for a baby with medical advice or a felt expectation to wait until optimally prepared for a subsequent pregnancy. While this research only included participants who went on to pursue a subsequent pregnancy, the short inter-pregnancy interval (median=7 months) is in keeping with research indicating a short inter-pregnancy interval after baby loss among WWD.^10 15^ It is crucial to consider the impact of a short inter-pregnancy interval in the context of diabetes for three main reasons: (1) the small window of opportunity to refer WWD to other services to support them in grieving the loss of their baby and preparing for a subsequent pregnancy; (2) a sensitive discussion about subsequent pregnancy may be welcomed earlier than healthcare professionals might assume and (3) it is important all healthcare professionals know about the benefits and requirements of preparing for pregnancy as WWD may come into contact with various healthcare professionals during the inter-pregnancy interval.

It is largely acknowledged that managing diabetes during pregnancy is challenging,^28 29^ which may be why so much support is available antenatally. However, women described how being 'optimally prepared' meant acting as if pregnant but with less support. WWD may feel overwhelmed or distressed by the prospect of being in 'pregnancy mode' for any longer than was necessary. It is plausible that the perceived lack of time to conceive and the burden of preparing for pregnancy contribute to the low uptake of pre-pregnancy care support and services in the inter-pregnancy interval. These findings highlight how WWD may require additional support during the inter-pregnancy interval, due to the mental health impact of baby loss^30^ and the additional psychosocial pressures WWD may experience during pregnancy.^31^ For example, WWD may experience heightened psychological and diabetes distress,^32 33^ although there is a paucity of research exploring the specific impact that baby loss has on this group’s mental and emotional health when becoming pregnant again.

The participants were distributed across the UK, highlighting differences in healthcare services delivery. The participants reported experiencing several gaps in care provision during the inter-pregnancy interval, with a lack of clear referral pathways. This finding is in keeping with previous research that described pre-pregnancy care as fractured, with a lack of agreement about who should provide pre-pregnancy care and the best way to deliver it.^11^ The participants with T2DM described difficulties in accessing care and support which could be attributed to the primary/secondary care divide, where the management of their diabetes was returned to primary care, but pre-pregnancy services were based in secondary care. This suggests preparing for pregnancy may be a particular challenge for those with T2DM, who are usually older when diagnosed,^34 35^ and who perhaps have not had opportunity for extended pregnancy-related discussions as they have less routine contact with healthcare professionals.^36^ It is vital that pregnancy is mentioned at or around the time of diagnosis, as women with T2DM may be taking teratogenic medications^4^ and may otherwise be reviewed infrequently. The ‘reproductive potential’ of women with T2DM must not be overlooked,^12 36^ as T2DM accounts for 55% of pregnancies to WWD and rates of serious outcomes for this group continue to increase.^4^ Although not all WWD will want to become pregnant again after a baby loss, many will become pregnant again soon after.^10^

Participants reported feeling that it was unclear who and when they were supposed to be talking to about pre-conception care for future pregnancy. There was no one service where all women with pre-existing diabetes could be wholly supported in the inter-pregnancy interval. The findings from a recent paper by the authors reporting the healthcare professionals’ views from the research study suggest that healthcare professionals may not think they are best placed to meet WWD’ needs and may assume other services provide support.^13^ These findings suggest that current approaches to pre-pregnancy care in the inter-pregnancy interval do not always meet the needs of this group. Namely, not all services were reported as having a dedicated pre-pregnancy care service for WWD, which means there may be a ‘postcode lottery’ for receiving support. Where pre-pregnancy care services did exist, the referral process was not always straightforward. It sometimes took a long time, which meant it risks being another burden for WWD if it was not easy to access in a timely way. Fundamental to improving the service to WWD is local services (diabetes, maternity, primary care, public health and commissioning teams) working collaboratively to create coordinated national initiatives^37^ that are flexible to meet the diverse needs of this group. Making recommendations that can be practically implemented remains a challenge in the context of constrained resources in the UK NHS system. A one-size-fits-all approach is unlikely to be effective in meeting the diverse needs of this group suggesting that more personalised, targeted support is needed in the inter-pregnancy interval. Future research is urgently required to address gaps in understanding, to better understand the psychosocial impact of baby loss on this group, and to address the widening health inequalities and disparities faced by women with T2DM (also a key priority in the recently published NPID Audit report).^4^ Research with a larger sample size that can provide the contextual details is needed to inform workable service development and work towards improving outcomes for these women.

### Strengths and limitations

This exploratory study focuses on a gap in the literature where there is little understanding to date on optimising inter-pregnancy care for WWD. Existing studies focus on pre-pregnancy care for WWD,^11 12 36^ and the challenges of navigating pregnancy after a baby loss.^38^ However, to our knowledge, this is the first study to bring these areas together with a focus on WWD’ lived experiences of the inter-pregnancy interval, despite this group being more likely to experience a baby loss. This paper represents the experiences of women with both T1DM and T2DM, who have experienced a range of early and late baby losses. Although the decision to include both T1DM and T2DM in this research posed a challenge as the conditions are different in terms of pathophysiology and aetiology, it was an opportunity to explore how the inter-pregnancy interval was experienced by participants and offered the potential to highlight differences in healthcare delivery.

In terms of limitations, there was no limit on time elapsed since the events women were reporting on, which could have impacted participant recollections, and care provision could have since changed. The WWD participants predominantly had T1DM (n=9), so the voices of women with T2DM (n=3) were underrepresented. As a small, exploratory study, our sample selection may necessarily be more reflective of the views of well-motivated people eager to emphasise issues experienced. These findings are not generalisable and do not aim to represent the views of all women in this situation. For example, women who did not pursue a subsequent pregnancy after baby loss were not eligible for inclusion owing to the risk of interviewing participants who were currently in the process of trying to conceive after a baby loss. The topic’s sensitive nature may have contributed to the small sample size, but this is in keeping with the sample size in other baby loss literature^39^ Recruiting and collecting the data online due to COVID-19 restrictions could have contributed to the homogeneity of the sample; most participants were white and well-educated. However, as an exploratory study of an under-researched area, women’s lived experiences are an important part of moving understandings forward, and these findings can help to inform larger studies in the future that aim to be more representative.

## Conclusions

These valuable findings shed light on some of the complex challenges experienced by WWD in the inter-pregnancy interval, an often over-shadowed part of the pregnancy journey. The focus on WWD’ lived experiences highlights that decisions around future pregnancy intention may happen soon after a baby loss, and sooner than healthcare professionals may assume. There may be a small window of opportunity to help WWD grieve for the baby loss and start preparing for a subsequent pregnancy. However, women might not feel comfortable initiating a conversation about subsequent pregnancy themselves.

Most participants found that being ‘optimally prepared’ for pregnancy was not straightforward and took a great deal of effort, both mentally and physically. On the whole, most participants found it challenging to navigate services and access pre-pregnancy care that was equipped to support them holistically. There were also apparent disparities between the accessibility and provision of care for women with T2DM compared with those with T1DM.

Overall, the findings suggest that a one-size-fits-all approach is unlikely to be effective in meeting the diverse needs of this group and that more personalised and targeted support is needed in the inter-pregnancy interval. The findings highlight the need for all healthcare professionals across the different parts of the care provision structure to work collaboratively, but flexibly to best meet the needs of WWD in the inter-pregnancy interval. Women with T1DM and T2DM may need to be targeted separately to ensure all WWD have timely access to appropriate pre-pregnancy care and support.

## Data Availability

Data are available upon reasonable request.
