# Supplementary material for: How can more women with pre-existing type 1 and type 2 diabetes be supported to prepare for pregnancy after a baby loss? A qualitative exploration of lived experiences in the UK
Source: BMJ Open. 2025 Jan 15;15(1):e083192. doi: 10.1136/bmjopen-2023-083192 (PMC11751928; doi:10.1136/bmjopen-2023-083192)
Supplement: Supplementary file 1 [file bmjopen-2023-083192.reviewer_comments.pdf]

PEER REVIEW HISTORY

BMJ Open publishes all reviews undertaken for accepted manuscripts. Reviewers are asked to complete a checklist review form and are provided with free text boxes to elaborate on their assessment. These free text comments are reproduced below.

ARTICLE DETAILS

Title (Provisional)

How can more women with pre-existing type 1 and type 2 diabetes be supported to prepare for pregnancy after a baby loss? A qualitative exploration of lived experiences in the United Kingdom

Authors

Dyer, Eleanor; Bell, Ruth; Graham, Ruth; Rankin, Judith

VERSION 1 - REVIEW

**Reviewer**	**1**
**Name**	**Hatunic, Mensud**
**Affiliation**	**Mater Misericordiae Hospitall, Dublin**
**Date**	**17-Jan-2024**
**COI**	**None**

This paper has high clinical importance. The paper is well written and easy to read.

I have minor recommendation: the recruitment was via social media and I feel that this is a small sample of well motivated patients who are eager to emphasize the issue with the inter-pregnancy interval after baby loss. The authors should add this in the discussion

---

**Reviewer**	**2**
**Name**	**Goodman, Jean**
**Affiliation**	**University of Missouri**
**Date**	**04-Feb-2024**
**COI**	**none**

I read with interest the submitted manuscript by Dyer et all, which reports on a qualitative assessment on a cohort of women with pre-gestational diabetes who experienced a perinatal loss and proceeded with another pregnancy.. The goal of the study was to define opportunities to improve outcomes in those particular future pregnancies in the inter-pregnancy period. The paper concludes women with diabetes may face challenges accessing

pre-pregnancy care and that a personalized and targeted support approach is needed. The conclusion is supported by a volume of literature published to date including from one of the authors of this submission, Murphy HR, Bell R, Dornhost A, Forde R, Lewis-Barne D. Pregnancy in Diabetes: Challenges and opportunities for improving pregnancy outcomes. N. Diabet Med. 2018 Mar;35(3):292-299. Unfortunately the study presented for publication suffers from many methodology concerns, particularly with regard to small subject volume and recall bias. Some examples of the design issues include the following: The study population is severely limited, with only 9 women with Type 1 diabetes and only 3 women with Type 2 diabetes. The study design was that of a semi-structured survey of women solicited through social media with only women with Type 1 or Type 2 diabetes, who had had a pregnancy-related loss and who pursued another pregnancy after that loss were interviewed. Therefore, we have no information from diabetic women who had a loss and elected not to pursue another pregnancy. There was no time limit from when the loss occurred, or the subsequent pregnancy occurred with respect to the interview timing – in some cases the interview was conducted many years later.. Per Table 1, we are not provided with demographics at the time of the loss or next pregnancy but rather at the time of the interview. Prior loss type was primarily early loss, and as described by the subject without validating medical records. The presumption is the losses were due solely to diabetes, but co-morbidities and thus potential confounders are not provided, nor are we provided information regarding chromosome or structural abnormalities which may have contributed to the loss(es). We are also not provided which pregnancy was affected by the loss – ie it does not appear the losses were the first pregnancy, based on descriptives within the paper or when multiparous which pregnancy was affected. We are also not provided the outcomes of the future pregnancies pursued.

Women who experience a perinatal loss and desire another pregnancy often wish to pursue that quickly regardless of etiology of the loss or co-morbidities, so the importance of post-loss and preconceptual care cannot be stressed enough. Add a co-morbidity like diabetes, and this urgency is further compounded. Redesign of the study to include a larger population, with a comparison group of those diabetics who choose not to pursue another pregnancy, assessment of co-morbidity impact, and comparison is suggested as that would provide much more robust data and the ability to draw more concrete conclusions to hopefully drive change to improve outcomes for these patients.

**Reviewer**	**3**
**Name**	**Benton, Madeleine**
**Affiliation**	**King's College London, Department of Psychological Medicine**
**Date**	**11-Aug-2024**

COI **NA**

Overall:

Thank you for inviting me to review this paper. I would like to congratulate the authors on completing this piece of work, it is a very valuable and important topic which has received limited attention. Overall, the manuscript provides value and important insight for the scientific and clinical community and my suggestions are to improve the overall piece of work.

However, I would like to highlight a key concern with the manuscript. I don't believe type 1 diabetes and type 2 diabetes should have been combined within one study, these are very different type of diabetes which are managed differently in pregnancy. There is also a flaw in that the authors did not include women with experienced gestational diabetes (which affects over 10% of pregnancies in the UK). The authors need to clearly justify why they made this decision. They will also need to pay careful consideration particularly in the introduction and discussion to highlight the key differences in care and experiences in pregnancy (e.g. stigma) and how this may have impacted their findings, and therefore recommendations.

Abstract:

Background:

The authors state "Although preparation and planning for pregnancy can reduce this heightened risk, only 50% of women with diabetes (WWD) do so, including after baby loss" - can this please be revised to improve readability? What do the authors means 50% after baby loss? Does this mean 50% of women with diabetes and who experience baby loss seek information for their next pregnancy?

Aims:

The aim is poorly worded for a qualitative study, I suggest re-working.

Methods:

There is a typo in "Qualitative data were collected November 2020-July 2021", 'between' seems to be missing before the dates. Please also add the word diabetes are types.

Results:

The authors state "thematic areas", I assume they mean themes, please correct.

Overall:

The authors use the term inter-pregnancy interval, a very short definition or explanation would be helpful for the general reader.

The authors use WWD, I would advise not to use this terminology as it is not common within the space.

Conclusion:

The authors conclude that women should receive individualised support and health providers need to be more aware, please provide key recommendations for research and/or practice, how could this be achieved within the NHS which is under considerable strain.

Introduction:

1. The authors state “Studies suggest 1-2% of pregnancies” – perhaps this is all diabetes cases combined but it is important to highlight that GDM is much more common – around 10%.

2. The authors state that the reason for increased prevalence in diabetes in pregnancy is because of “both the rise in overweight and obesity rates in the general population” this is a good point for type 2 diabetes but not for type 1 diabetes.

3. The authors use the terms ‘trebling’ and ‘quadrupling’ when discussing increases in risk, can they please provide figures.

4. The authors state that the reasons for women with diabetes not being prepared for pregnancy are ‘complex’ but provides no reference of review of the limited published research. Please amend the introduction to accommodate this. There is also no inclusion of the psychological literature of diabetes in pregnancy - there are several qualitative pieces of work which highlight increased stress and worry around baby loss for women with diabetes, yet this hasn't been included.

5. As highlight previously, I would like to see some background to T1D and T2D.

6. There is also missing details as to what current care is in the UK after baby loss for women with diabetes – if any.

Methods:

1. Can the authors please justify why there was no PPIE conducted? Considering this is such a sensitive topic I would have at a minimum expected women to be consulted about the topic guide.

2. The authors state “ED” conducted the interviews please change to “first authors (ED)”.

3. The authors state “The anonymised transcripts formed the data for the qualitative analysis” however, these is not detail of how the interviews were transcribed, what methods was used and by whom e.g. verbatim?

4. There is no detail about the steps without RTA, please include.

5. Detail of positionality is missing from the methods.

Results

1. The themes and sub themes are clear.

2. I advise the authors to do another read through of their analytic bridges to ensure they are clear and concise without repetition.

3. The authors use ellipsis within the quotes '…' but have put them in square brackets which isn't correct. Please remove square brackets, there should be a space before and after the 3 periods.

4. There are 2 figures which relate to the results included in the appendix, these are not labelled and not properly discussed in text.

Discussion

1. The authors have presented more of a summary of their findings rather than property discussing them in the context of previous re related research. Please revise.

2. As the study was based in the UK, it is important to provide more detail of the local context and recommendations for the NHS and then they can be discussed in more of an international context.

3. Limitations – please include the limitations of combining women's experience of T1D and T2D and not including women with GDM.

VERSION 1 - AUTHOR RESPONSE

Reviewer 1 Comments

Dr. Mensud Hatunic, Mater Misericordiae Hospital, Dublin

This paper has high clinical importance. The paper is well written and easy to read.

Reviewer 1 Comments	Authors Response	Line number(s) in marked copy
I have minor recommendation: the recruitment was via social media and I feel that this is a small sample of well motivated patients who are eager to emphasize the issue with the inter-pregnancy interval after baby loss. The authors should add this in the discussion.	Thank you for this comment. It was necessary to use social media to recruit participants as data collection took place during the height of the COVID-19 pandemic. We agree that this approach has its limitations, and as such, we have added a sentence to reflect your comment in the discussion section along with other limitations.	605-607

Reviewer 2 Comments

Prof. Jean Goodman, University of Missouri
Comments to the Author:
I read with interest the submitted manuscript by Dyer et all, which reports on a qualitative assessment on a cohort of women with pre-gestational diabetes who experienced a perinatal loss and proceeded with another pregnancy. The goal of the study was to define opportunities to improve outcomes in those particular future pregnancies in the inter-pregnancy period. The paper concludes women with diabetes may face challenges accessing pre-pregnancy care and that a personalized and targeted support approach is needed. The conclusion is supported by a volume of literature published to date including from one of the authors of this submission, Murphy HR, Bell R, Dornhost A, Forde R, Lewis-Barne D. Pregnancy in Diabetes: Challenges and opportunities for improving pregnancy outcomes. N. Diabet Med. 2018 Mar;35(3):292-299.

Reviewer 2 Comments	Authors Response	Line number in marked copy
Unfortunately the study presented for publication suffers from many methodology concerns, particularly with regard to small subject volume and recall bias. Some examples of the design issues include the following: The study population is severely limited, with only 9 women with Type 1 diabetes and only 3 women with Type 2 diabetes.	Thank you for your comments and feedback. As a PhD project, this research study was restricted in time and resource and the COVID-19 pandemic posed a significant challenge to recruitment as the study was conducted over the course of multiple national lockdowns. However, as the analytic focus was on understanding women's lived experiences, a smaller sample size permitted a more in-depth qualitative analysis of their accounts.<br><br>We also conducted interviews with 18 healthcare professionals as part of the wider PhD research study (30 participants in total), however, due to wordcount restrictions, we decided to report these findings separately. We have made it more explicit in the design and acknowledgements sections that this research was part of a larger study.<br><br>We acknowledge that the sample size of women with diabetes was small and a limitation of the study. However, as an exploratory study, we feel it offers rich and nuanced insight into participants' lived experiences, and adds to knowledge about women's	154-160; 683-684

Reviewer 2 Comments	Authors Response	Line number in marked copy
	accounts in an underdeveloped area in the literature. As such, this work will contribute to the evidence base in the future.	
The study design was that of a semi-structured survey of women solicited through social media with only women with Type 1 or Type 2 diabetes, who had had a pregnancy-related loss and who pursued another pregnancy after that loss were interviewed. Therefore, we have no information from diabetic women who had a loss and elected not to pursue another pregnancy.	Thank you for this comment. We acknowledge that not every woman with diabetes will want to pursue another pregnancy, and we were clear about this in the strengths and limitations section and in the discussion section.<br><br>Our justification for focussing only on women who pursued a subsequent pregnancy were twofold: 1) previous studies have shown that many women with diabetes who experienced a baby loss would want to become pregnant again soon after the loss. We wanted to explore what is was like for this group of women between pregnancies, especially given that we know that being ‘optimally prepared’ can help reduce the risk of baby loss, so there is a clear opportunity here to improve or encourage pre-pregnancy care; 2) we only included women who had gone on to ‘complete’ a subsequent pregnancy to try and mitigate the risk of causing undue harm and distress by interviewing women who were currently trying to conceive after a baby loss.	103-104;<br>554-556;<br>609-611
There was no time limit from when the loss occurred, or the subsequent pregnancy occurred with respect to the interview timing – in some cases the interview was conducted many years later.	Thank you for this comment. We acknowledged that there was no limit on time elapsed since the events women were reporting on, which could have impacted participant recollections, and care provision could have since changed in the limitation section.<br><br>There were three main reasons we decided not to put a limit on the time	96-98;<br>601-603

Reviewer 2 Comments	Authors Response	Line number in marked copy
	elapsed: 1) We were recruiting for a niche group as part of an exploratory study, during challenging COVID-19 circumstances. We did not want to put too many restrictions on who would be eligible, rather we sought to recruit as diverse a range of experience as possible; 2) Following advice from our collaborators, Sands, (a national charity specialising in baby loss) we felt it was important to honour women's experiences and not diminish them by suggesting that something that happened a long time ago no longer mattered; 3) in some cases it was important that enough time had elapsed for the women to share their experiences without becoming re-traumatised by revisiting the events discussed.	
Per Table 1, we are not provided with demographics at the time of the loss or next pregnancy but rather at the time of the interview.	Thank you for your comment. For reasons of anonymity, it is not possible to share more detailed information about the demographics of the participants. As our analytic focus was on women's accounts of their experiences, the most relevant details (at the time of interview) have been shared. From a social science perspective, the ways that women remember their experiences are also important to our understandings of how to enhance care provision from a service user perspective.	N/A
Prior loss type was primarily early loss, and as described by the subject without validating medical records.	We disagree that prior loss type was primarily early loss. We interviewed 12 women, four of whom had experienced multiple losses. Prior losses included early miscarriage (8) and late miscarriages (5), as well as stillbirths (2) and neonatal deaths (3). Furthermore, we do not wish to suggest that early losses are any less	N/A

Reviewer 2 Comments	Authors Response	Line number in marked copy
	significant, and so do not see this as a limitation.<br><br>We feel it was not possible or necessary, considering the scope of this research project, to validate losses against medical records. It is not possible to provide more detailed information about each individual participant as this could compromise their anonymity. As noted above, our analysis focused on women's lived experiences and how these were remembered. Whilst comparison with formally recorded medical information might have gleaned insights, these were not the focus of our analysis and did not have ethical approval for this.	
The presumption is the losses were due solely to diabetes, but co-morbidities and thus potential confounders are not provided, nor are we provided information regarding chromosome or structural abnormalities which may have contributed to the loss(es).	Thank you for this comment. It was not our intention to suggest that the losses were attributed solely to diabetes, and the purpose of this research was not to report on the cause of the loss, but to explore the experiences of this group of women who were affected by a loss. As our analytic focus was on women's lived experiences of preparing for pregnancy after loss, the specific circumstances of the loss were not the focus of the analysis.<br><br>We were interested in how women with diabetes can be better supported to prepare for a pregnancy after a loss, given that being 'optimally prepared' for pregnancy could reduce the risk of further baby loss. We wanted to understand the specific challenges faced by this group to find out how participants felt that healthcare services could better meet their needs.	N/A

Reviewer 2 Comments	Authors Response	Line number in marked copy
We are also not provided which pregnancy was affected by the loss – ie it does not appear the losses were the first pregnancy, based on descriptives within the paper or when multiparous which pregnancy was affected. We are also not provided the outcomes of the future pregnancies pursued.	For reasons of anonymity, we are unable to share more granular data about the participants' experiences of baby loss. We discussed this comment and feel like this information is not directly relevant to the aims of this paper given it's exploratory approach and focus on women's lived experiences. However, we note that it would be potentially useful to gather this type of information in future for a study that focused on such aspects of loss.	N/A
Women who experience a perinatal loss and desire another pregnancy often wish to pursue that quickly regardless of etiology of the loss or co-morbidities, so the importance of post-loss and preconceptual care cannot be stressed enough. Add a co-morbidity like diabetes, and this urgency is further compounded. Redesign of the study to include a larger population, with a comparison group of those diabetics who choose not to pursue another pregnancy, assessment of co-morbidity impact, and comparison is suggested as that would provide much more robust data and the ability to draw more concrete conclusions to hopefully drive change to improve outcomes for these patients.	Thank you for this comment. We agree with your summary with regard to the importance of this topic and offering post-loss and pre-pregnancy care.<br><br>We agree that future research into this area is urgently required with a larger sample size that can provide the contextual details needed to draw more concrete conclusions, inform workable service development, and hopefully drive change to improve outcomes for these patients. We have made this more explicit in the discussion section.<br><br>Nevertheless, exploratory studies like this are an important first step, and although there is a small sample size, the rich and nuanced data provide important insight into this under-researched area.	580-583

Reviewer 3 Comments

Dr. Madeleine Benton, King's College London
Comments to the Author: Thank you for inviting me to review this paper. I would like to congratulate the authors on completing this piece of work, it is a very valuable and important topic which has received limited attention. Overall, the manuscript provides value and important insight for the scientific and clinical community and my suggestions are to improve the overall piece of work.

Reviewer 3 Comments	Authors Response	Line number in marked copy
However, I would like to highlight a key concern with the manuscript. I don't believe type 1 diabetes and type 2 diabetes should have been combined within one study, these are very different type of diabetes which are managed differently in pregnancy.	Thank you for this comment. We agree that the pathophysiology and aetiology of the conditions are distinct. However, as the focus of this research was on women's experiences of baby loss and preparing for subsequent pregnancy, it was important to include both types of diabetes because they are associated with similar rates of baby loss. The NICE guidelines for 'optimal' pregnancy preparation are predominantly aimed at type 1 and type 2 diabetes, and both conditions are the focus of the National Pregnancy in Diabetes Audit.<br><br>Whilst the decision to include both type 1 and type 2 diabetes in this research posed a challenge in terms of the conditions being very different, it was also an opportunity to explore how the inter-pregnancy interval was experienced by participants, and offered the potential to highlight differences in healthcare delivery, especially with regards to the recommendations for women with both type 1 and type 2 pregnancy to prepare for pregnancy.	92-95;<br>592-600
There is also a flaw in that the authors did not include women with experienced gestational diabetes (which affects over 10% of pregnancies in the UK). The authors need to clearly justify why they made this decision.	Thank you for this comment. We appreciate that including women with experience of gestational diabetes may have been an interesting avenue to pursue. However, it was beyond the scope of this research given that the focus was on how women can be supported to prepare for a pregnancy in the context of diabetes; as gestational diabetes usually resolves after delivery (Plows et al., 2018), the advice and care offered to this group is significantly different to the care packages aimed at women with type 1 and type 2 diabetes, especially with regards to the advice surrounding	

	preparing for pregnancy. That being said, we agree that this is an interesting idea and would be keen to explore women's experience of gestational diabetes in future research, as we build on this exploratory work.	
They will also need to pay careful consideration particularly in the introduction and discussion to highlight the key differences in care and experiences in pregnancy (e.g. stigma) and how this may have impacted their findings, and therefore recommendations.	We agree that stigma is an important factor in care. However, given the nature of this exploratory study, and the journal's word limit, we have not been able to include all aspects of care that could be considered. Instead, we have focused our analysis on the descriptive themes that were generated during the analysis. We plan to report separately on WWD's experiences of stigma during the inter-pregnancy interval, as we explored this topic in depth in the broader project and the wordcount constraints here mean that we are unable to cover this aspect in sufficient depth to do the data and the participants justice.	N/A
Abstract:<br>Background:<br>The authors state "Although preparation and planning for pregnancy can reduce this heightened risk, only 50% of women with diabetes (WWD) do so, including after baby loss" - can this please be revised to improve readability? What do the authors means 50% after baby loss? Does this mean 50% of women with diabetes and who experience baby loss seek information for their next pregnancy?	Thank you for this comment, we agree that there was room for improvement, and we have updated the manuscript to make this clearer.	133-134
Abstract Aims:<br>The aim is poorly worded for a qualitative study, I suggest re-working.	We have amended and reworded the abstract following the Editor's comments.	38-65
Abstract Methods:<br>There is a typo in "Qualitative data were collected November 2020-July	As above, the abstract has been reworked following the Editor's comments.	38-65

2021", 'between' seems to be missing before the dates. Please also add the word diabetes are types.		
Abstract Results:<br>The authors state "thematic areas", I assume they mean themes, please correct.	Thank you for this observation, we have amended this and agree that it reads better.	50
Overall:<br>The authors use the term inter-pregnancy interval, a very short definition or explanation would be helpful for the general reader.	Thank you for this comment. We have added a description to the abstract and another description is provided in the methods section.	39-40;<br>187
Overall: The authors use WWD, I would advise not to use this terminology as it is not common within the space.	Thank you for this comment. We agree that the overuse of acronyms can have a negative impact on readability and so have kept acronyms to a minimum. However, we were also struggling to meet the wordcount limits set by the journal, and were required to use first-person language, as per the Language Matters guidance (NHS, 2018). Although we recognise that person first language such as 'women with diabetes' is a contested area, it is currently the preferred way to address this group as labelling a person as a 'diabetic' can be perceived as stigmatising (NHS, 2018). As such, using WWD was actually preferable to spelling out 'women with diabetes' every time in terms of readability.	N/A
Abstract Conclusion:<br>The authors conclude that women should receive individualised support and health providers need to be more aware, please provide key recommendations for research and/or practice, how could this be achieved within the NHS which is under considerable strain.	These are important concerns, particularly given recent social commentary about the NHS in the UK media. Whilst it is beyond the scope of this exploratory study to suggest that we know all the answers to solve these problems, especially within the limits of what is achievable within the abstract, we agree that it would be useful to flag this wider health policy context in our discussion, and the challenges of making recommendations that can be practically	572-574

	implemented in a context of constrained resources.	
Introduction:<br>1. The authors state "Studies suggest 1-2% of pregnancies" – perhaps this is all diabetes cases combined but it is important to highlight that GDM is much more common – around 10%.	We do not feel that including GDM is relevant in this study that is exploring the experiences of women with type 1 and type 2 diabetes, given these groups are encouraged to plan and prepare for pregnancy to reduce risks. We discussed adding a statistic about GDM, but have decided against it as we are restricted by the wordcount and number of references allowed and felt that mentioning GDM would risk confusing the reader as we do not have the wordcount nor references to elaborate. We have, however, reworded the beginning of the background section to make it more explicit that this research refers to pre-existing type 1 and type 2 diabetes.	109-111
Introduction:<br>2. The authors state that the reason for increased prevalence in diabetes in pregnancy is because of "both the rise in overweight and obesity rates in the general population" this is a good point for type 2 diabetes but not for type 1 diabetes.	Thank you for this comment. Weight gain and body mass are related to the onset of both type 1 and type 2 diabetes (Al-Goblan, Al-Alfi and Khan, 2014), although the link is possibly clearer for type 2 diabetes, and we have added clarification about that to our text.	113
Introduction:<br>3. The authors use the terms 'trebling' and 'quadrupling' when discussing increases in risk, can they please provide figures.	We have amended this section as per your comment to improve readability and freeup wordcount and references for other comments.	115-120
Introduction:<br>4. The authors state that the reasons for women with diabetes not being prepared for pregnancy are 'complex' but provides no reference of review of the limited published research. Please amend the introduction to accommodate this.	Thank you for this comment. We feel it is important to highlight that there are multifactorial reasons why women with diabetes might not prepare for a pregnancy, as it is not easy to achieve 'optimal' preparation as set out in the NICE NG3 preconception guidelines. However, we were restricted here by the journal's wordcount and number of references allowed (40). We have strengthened this section by rewording	132

	slightly to highlight that there is limited published research in this area – a gap that this paper attempts to bridge.	
There is also no inclusion of the psychological literature of diabetes in pregnancy - there are several qualitative pieces of work which highlight increased stress and worry around baby loss for women with diabetes, yet this hasn't been included	Thank you for highlighting this omission. We are aware of this literature but as above, we were restricted here by the journal's wordcount and number of references allowed (40) and have strengthened the discussion section to include supporting literature on the psychological impact of diabetes, pregnancy and baby loss.	530-536
INtroduction:<br>5. As highlight previously, I would like to see some background to T1D and T2D.	As per above responses, we were restricted here by the wordcount and number of references allowed (40). We have, however, strengthened this section by reworking and rewording in response to previous comments and have added a sentence by way of providing more background.	115-129
Introduction:<br>6. There is also missing details as to what current care is in the UK after baby loss for women with diabetes – if any	Thank you for highlighting this omission. We have added a sentence to the background section to highlight that there is no standardised care in the inter-pregnancy interval.	127-129
Methods:<br>1. Can the authors please justify why there was no PPIE conducted? Considering this is such a sensitive topic I would have at a minimum expected women to be consulted about the topic guide.	Thank you for this comment and highlighting this error, as there was PPIE involvement in the design and conduct of this research. We have reworded this section to reflect these activities. For example, to ensure the interviews explored issues that were relevant to women with diabetes who had experienced a baby loss, the Stillbirth and Neonatal Death Charity, SANDS, were able to advise on the content to ensure terminology and questions were phrased sensitively.<br><br>Three women with lived experience of diabetes and baby loss were also involved in commenting on the topic guide and piloting the topic guides for the interviews	170-176

	with WWD which gave us confidence that the research topic was relevant and needed, and that the sensitive nature of the topic was handled appropriately. Since the completion of the project, PPIE involvement has been central to ongoing dissemination activities. We have amended the PPIE section to reflect this.<br><br>Further PPIE work with women would have been advantageous but was not possible given that this was a PhD project, this research study was restricted in time and resource and the COVID-19 pandemic posed a significant challenge to this research as the study was conducted over the course of multiple national lockdowns	
Methods:<br>2. The authors state "ED" conducted the interviews please change to "first authors (ED)".	Thank you for this comment, which we have addressed.	182
Methods:<br>3. The authors state "The anonymised transcripts formed the data for the qualitative analysis" however, these is not detail of how the interviews were transcribed, what methods was used and by whom e.g. verbatim?	Thank you for this comment, we agree that this sentence is a bit vague and so have provided more detail on the specific methods used. i.e. Interviews were transcribed verbatim, using manual methods, by the first author (ED) and a clerical officer. The transcripts were anonymised and then uploaded to Quirkos software for analysis.	198-199
METHODS:<br><br>4. There is no detail about the steps without RTA, please include.	Given the significant challenge of the wordcount, we have included a supplementary figure (Figure 1) to provide more detail about the analytic process, which is supported by a couple of sentences to explain the main premise of an RTA approach.	200-204
METHODS:<br>5. Detail of positionality is missing from the methods.	As above, the couple of sentences added as part of RTA explanation addresses this point. We included details about the theoretical approach (social constructionism) highlighting the interpretive nature of the findings along with references for the reader to follow	202-204;<br>208-215

	should they want to find out more. We are limited by wordcount and so do not feel this requires more explanation in the context of a journal article.	
Results<br>1. The themes and sub themes are clear.		
Results:<br>2. I advise the authors to do another read through of their analytic bridges to ensure they are clear and concise without repetition.	We have discussed this point and a thorough read through has been done and we have no amends. We are less familmiar with the 'analytic bridge' nomenclature and have only previously seen it in relation to the 'bridging' of qualitative and quantitative findings. If the reviewer is able to provide a specific reference to clarify their usage of the terminology, we would be happy to look into this further if needed.	
Results:<br>3. The authors use ellipsis within the quotes '…' but have put them in square brackets which isn't correct. Please remove square brackets, there should be a space before and after the 3 periods.	Thank you for this suggestion. We have removed the square brackets throughout the manuscript.	
Results:<br>4. There are 2 figures which relate to the results included in the appendix, these are not labelled and not properly discussed in text.	Thank you for this comment. The figures are now labelled and referred to in the main body of the paper. We are conscious that wordcount is limited and so after some discussion we agreed that the figures are self-explanatory and do not require additional discussion in the text.	201-202;<br>226
Discussion<br>1. The authors have presented more of a summary of their findings rather than property discussing them in the context of previous re related research. Please revise.	We followed the author guidelines when writing the discussion section and this section has been revised since addressing the above reviewer comments. For example, we have made clearer signposts to related research, albeit there is a gap in the literature in terms of studies focusing on this specific topic. We have also added a subheading to help separate out the strengths and limitations which we think improves the readability of this section.	532-536;<br>540-543;<br>587-618
Discussion:<br>2. As the study was based in the UK, it		572-577

is important to provide more detail of the local context and recommendations for the NHS and then they can be discussed in more of an international context.	Thank you for this comment and suggestion. We have reworked the discussion section to incorporate more context.	
Discussion:<br>3. Limitations – please include the limitations of combining women's experience of T1D and T2D and not including women with GDM.	As above, we have separated out the strengths and limitations section so that this section is more explicit.<br><br>In addition to this, and in response to the Editor's above request, a summary of the strengths and limitations is also provided toward the beginning of the article.	517-618;<br>596-600;<br><br>92-96
